# Regulatory roles of long non-coding RNAs in short-term heat stress in adult worker bees

**DOI:** 10.1186/s12864-024-10399-8

**Published:** 2024-05-22

**Authors:** Bing Zhang, Chaoying Zhang, Jiangchao Zhang, Surong Lu, Huiting Zhao, Yusuo Jiang, Weihua Ma

**Affiliations:** 1 College of Animal Science, Shanxi Agricultural University, Jinzhong, Shanxi China; 2 College of Life Sciences, Shanxi Agricultural University, Jinzhong, Shanxi China; 3 College of Horticulture, Shanxi Agricultural University, Taiyuan, Shanxi China

**Keywords:** *Apis mellifera*, Cis-regulation, Heat stress, lncRNA, *MSTRG.9645.5*, Network regulation, Trans-regulation

## Abstract

Long non-coding RNAs (lncRNAs) are crucial modulators of post-transcriptional gene expression regulation, cell fate determination, and disease development. However, lncRNA functions during short-term heat stress in adult worker bees are poorly understood. Here, we performed deep sequencing and bioinformatic analyses of honeybee lncRNAs. RNA interference was performed by using siRNA targeting the most highly expressed lncRNA. The silencing effect on lncRNA and the relative expression levels of seven heat shock protein (HSP) genes, were subsequently examined. Overall, 7,842 lncRNAs and 115 differentially expressed lncRNAs (DELs) were identified in adult worker bees following heat stress exposure. Structural analysis revealed that the overall expression abundance, length of transcripts, exon number, and open reading frames of lncRNAs were lower than those of mRNAs. GO analysis revealed that the target genes were mainly involved in “metabolism,” “protein folding,” “response to stress,” and “signal transduction” pathways. KEGG analysis indicated that the “protein processing in endoplasmic reticulum” and “longevity regulating pathway-multiple species” pathways were most enriched. Quantitative real-time polymerase chain reaction (qRT-PCR) detection of the selected DELs confirmed the reliability of the sequencing data. Moreover, the siRNA experiment indicated that feeding siRNA yielded a silencing efficiency of 77.51% for lncRNA *MSTRG.9645.5*. Upon silencing this lncRNA, the expression levels of three HSP genes were significantly downregulated (*p* < 0.05), whereas those of three other HSP genes were significantly upregulated (*p* < 0.05). Our results provide a new perspective for understanding the regulatory mechanisms of lncRNAs in adult worker bees under short-term heat stress.

**Supplementary Information:**

The online version contains supplementary material available at 10.1186/s12864-024-10399-8.

## Introduction

Insect pollinators, including honeybees, play crucial roles in maintaining ecosystem stability and biodiversity [1–3]. *Apis mellifera*, an extensively studied insect, has garnered significant attention because of its environmental and economic importance [4, 5]. However, bees face increasing survival pressures due to global climate change and human activities. Heat stress is one of the main causes of bee death [6]. Heat stress can disrupt the metabolism and physiological functions of bees, thereby affecting their survival and reproduction [5].

Long non-coding RNAs (lncRNAs) are RNA molecules longer than 200 nucleotides. They lack protein-coding ability. However, they are crucial in alternative splicing, cell cycle, epigenetics, dosage compensation, and gene expression regulation [7, 8]. LncRNAs are typically expressed at low levels and exhibit limited conservation across species [9]. Evidence indicates that lncRNAs containing miRNA-response elements (MREs) can regulate downstream target gene expression by absorbing miRNAs, thereby regulating specific biological processes [10]. This mechanism, known as competing endogenous RNA (ceRNA), has been confirmed in various species, including humans [11, 12], mice (Mus musculus) [13], and other species. However, research on ceRNA mechanisms in honeybees is currently lacking. Additionally, lncRNAs influence the expression of neighboring genes through two mechanisms: cis-regulation and trans-regulation [14].

Recently, lncRNAs roles among insect communities has garnered interest [15]. LncRNAs in various species, such as *Plutella xylostella* [16], *Apis cerana* [17], and *Ascospheara apis* [18] have been reported. With high-throughput sequencing technology and bioinformatics, lncRNAs have been identified in bees [19, 20]. Several studies have demonstrated the pivotal roles of lncRNAs in the growth [21], development [20], and caste differentiation [22] of honeybees. Currently, research on the function and mechanism of lncRNA is mainly focused on a few model organisms such as humans [11–13]. Although the transcriptomic analysis of honeybees provides insights into potential gene regulatory networks [17–19], only a few specific genes exhibit functional characteristics [23]. The functional aspects and mechanisms of lncRNA in bees remain largely unknown.

To survive better in extreme environments, bees deploy compensatory strategies at the behavioral and molecular levels to resist heat stress [5]. In terms of behavior, bees exhibit various actions such as fanning [24], water collection [25], and clustering to regulate the temperature inside the hive [26]. At the molecular level, high temperature induces the expression of several key genes and proteins, such as heat shock proteins (HSPs) [27, 28], nuclear factors (NF) [29], acetylcholinesterase 1 (AchE1) [30], serine/threonine protein kinases (STKs) [28]. These genes are involved in various biological processes such as cellular metabolism, protein folding, and degradation [31]. Among numerous thermal-resistance genes, the HSP gene family plays an extremely important role [5, 27, 28].

Using RNA sequencing, we sought to identify and analyze lncRNAs in adult worker bees that had been exposed to short-term heat stress. The potential regulatory mechanisms of these lncRNAs were explored by functional annotation and enrichment analyses of their target genes. Additionally, we conducted an experiment in which siRNA was administered to silence the most highly expressed lncRNAs, and the effect on the expression of HSPs genes was measured. These results provide a new insight into the roles of lncRNAs in the response to heat stress of honeybees and form the basis for further investigation of gene regulation in adult worker bees.

## Materials and methods

### Experimental insects

*Apis mellifera ligustica* was obtained from an experimental apiary at Shanxi Agricultural University (Taigu, China). Three naturally mated, healthy bee colonies were utilized in this study. Immediately before larva pupation, the brood combs were removed and placed in a controlled environment with constant temperature and humidity. The combs were incubated overnight at 34 ± 0.5℃ and 75 ± 5% relative humidity (RH). The following day, the honeybees were marked on the thorax using non-toxic and odorless dyes to designate them as 1-day-old (1d) bees, after which they were returned to their respective colonies. The marked bees were collected as samples at 20-days-old (20 d) and were used as foragers.

### Sample collection

Honeybees were collected at 20 d for further experiments. They were divided into two groups based on temperature treatments: normal conditions (25 ± 0.2℃, RH 30%, designated as CK) and high-temperature conditions (45 ± 0.2℃, RH 30%, designated as HT). Temperature selection was based on previous research conducted in the laboratory [32]. Each temperature group provided three biological replicates (ten bees × two temperature treatments × three replicates). The honeybees were subjected to their respective temperature conditions for two hours, then rapidly frozen using liquid nitrogen and stored at -80℃ for RNA isolation, library preparation, sequence, and validation of differentially expressed lncRNAs (DELs).

In addition to heat exposure, we performed RNA interference experiments. For this, 20-day-old honeybees were collected and housed in containers with specific dimensions (weight: 12.4 cm, height: 3.7 cm, depth: 3.2 cm). These containers were placed in an environmental incubator within a specified temperature range for 5 days. Following this period, the honeybees were divided into four groups, consisting of 60 individuals per group.

The diets for the different groups included negative interference fragments (siNC), interference fragment 1 (siRNA1), siRNA2, and siRNA3 (see Sect. 2.4.2 and 2.4.3). Throughout the experimental period, all groups were also fed sugar water (a ratio of 3:1 sugar to ddH2O). These bees were collected at 24 h intervals, flash-frozen using liquid nitrogen, and stored at -80℃ until used.

### LncRNA bioinformatics analysis

#### RNA isolation, library preparation, and sequencing

Total RNA was extracted from each sample using TRIzol™ reagent according to the manufacturer’s instructions (Ambion, Foster City, CA, USA). Next, ribosomal RNA (rRNA) was removed using a Ribo-off rRNA Depletion (Animal) Kit (Vazyme Biotech Co., Ltd., Nanjing, China) to enhance the sequencing coverage of the non-rRNA RNA. After removing the rRNA, we use a Hieff NGS® Ultima™ Dual-mode mRNA Library Prep Kit for Illumina (NEB, USA) to construct sequencing libraries. The RNA was then transcribed into complementary DNA (cDNA) using reverse transcriptase. Subsequently, the NEB-Next Adaptor was ligated for hybridization and the products were purified using the AMPure XP system (Beckman Coulter, Beverly, USA). Finally, the library quality was assessed using Qsep-400, and high-throughput sequencing was performed on the Novaseq 6000 (Illumina, San Diego, CA, USA) using a HiSeq Rapid SBS sequencing kit version 2 to produce 300 bp paired-end sequences. This resulted in 318,998,995 read pairs (93.97 Gb of raw data).

#### Read mapping and lncRNA identification

Clean reads were obtained by removing adapters, low-quality reads (quality scores < 20), and poly-N (with a ratio of “N” > 10%) from raw data. Quality control (QC) calculations (Q20, Q30, and GC) were performed simultaneously by FastQC. High-quality clean reads were used for subsequent analyses. Clean reads were mapped to the honeybee reference genome [33] (Amel_OGSv3.2, https://hymenoptera.elsiklab.missouri.edu/ogs_gff3_files) using HISAT2 [34]. The mapped reads were merged using the StringTie software [35].

Bioinformatic predictions included basic screening and potential coding screening. Transcript sequence information was obtained through basic screening. Transcripts with class codes “i,” “x,” “u,” “o,” and “e,” were selected as novel long transcripts [36]. Transcripts with more than two exons and exceeding 200 bp in length were further analyzed to identify lncRNAs [37]. The transcripts obtained were compared to annotation databases, including the Coding Potential Calculator (CPC) [38], Coding-Non-Coding Index (CNCI) [39], Coding Potential Assessment Tool (CPAT) [40], and Pfam [41].

#### Analysis of DE lncRNAs

StringTie is an algorithm based on the optimization theory used to calculate fragments per kilobase of exon model per million (FPKMs) for each fragment [35]. We integrate the output results from StringTie with DESeq2 using a model based on the negative binomial distribution to identify differential expression analysis. Genes with |fold change| ≥ 2 and FDR < 0.05 were set as the thresholds for DELs.

#### Prediction, functional enrichment, and interaction network construction

Based on the mode of interaction between lncRNAs and their target genes, we used two prediction methods. First, lncRNAs regulate the expression of nearby genes, predicted based on the positional relationship between the lncRNAs and target genes [42]. Genes within 100 kb of the lncRNAs were considered cis-target genes [43]. Genome annotation and browsers were used to identify possible target genes of the lncRNAs. A Perl script was used for selection. The second method predicted the trans-target genes of lncRNAs through a correlation analysis of lncRNA and mRNA expression between samples. The Pearson correlation coefficient (PCC) method was used to analyze correlations. Genes with PCC > 0.9 and *p*-value < 0.01 were selected as lncRNA trans-target genes.

Gene Ontology (GO) enrichment analysis of the target genes of DELs was performed using the GOseq R package (https://bioconductor.org/packages/release/bioc/html/goseq). KOBAS (v2.0; https://bio.tools/kobas) was used to test the statistical enrichment of DE genes (DEGs) or target genes of lncRNAs in KEGG pathways. *P*-values < 0.05 were considered to indicate significant enrichment.

To identify critical lncRNAs associated with heat tolerance, an interaction network comprising DELs and mRNAs was constructed using Gephi (v0.8.2; https://gephi.org/) software based on cis- or trans-regulation.

#### Validation of DELs

Quantitative real-time polymerase chain reaction (qRT-PCR) was performed to verify the accuracy of the RNA-seq. Ten genes were randomly selected for verification. TRIzol™ reagent (Invitrogen, Carlsbad, CA, USA) was used to extract RNA according to the manufacturer’s instructions. RNA was reverse transcribed to cDNA using a reverse transcription kit (Takara Co., Ltd., Dalian, China). qRT-PCR was performed using a Bio-Rad CFX96 with a SYBR Premix ExTaq™ kit (Takara Bio, Inc.) according to the manufacturer’s instructions. Primers were synthesized by Shanghai Sangon Co., Ltd. (Shanghai, China), and these sequences can be found in Supplementary Table 1. Gene expression was quantified relative to the expression of *ACTB* using the comparative cycle threshold (ΔCT) method. Each sample was run in triplicate.

### Interference

#### Subcellular localization prediction and target analysis of MSTRG.9645.5

The online tool LncLocator [44] (http://www.csbio.sjtu.edu.cn/bioinf/lncLocator/) was used to predict the subcellular localization of *MSTRG.9645.5*. Miranda and TargetScan software [45] were used to predict the miRNA targeted by *MSTRG.9645.5* and the mRNA targeted by the miRNA. The regulatory network of *MSTRG.9645.5* was visualized using Cytoscape software [46].

#### Design and synthesis of siRNA

The lncRNA with the highest expression level was selected from the transcriptome data. Its secondary structure, conserved structural motifs, and possible domains or structural domains were predicted using VNTI software. Based on the above predicted results and the gene sequences of the selected lncRNAs, the specific target small interfering RNA was designed. Three siRNA sequences targeting the lncRNA and one control sequence were selected. Double-stranded siRNAs were synthesized by Synbio Technologies (Shanghai) Co., Ltd, and these sequences can be found in Supplementary Table 2.

#### Rearing of bees

Four randomly divided bee groups were individually fed different substances: siNC group (control group targeting non-transcribed or scrambled sequences), siRNA1 group, siRNA2 group, and siRNA3 group. Each bee was fed 10 µL of the feeding solution, containing a siRNA concentration of 10 ng/µL. After feeding, the bees were reared in an artificial climate chamber and provided with sugar water during the experiment. Bee samples were collected at 24, 48, 72, 96, and 120 h after feeding. Ten bees from each treatment group were randomly selected at each time-point. Immediately after collection, bee samples were transferred to liquid nitrogen and stored at -80 °C for further analysis. Next, qRT-PCR was used to detect the optimal interfering fragment and time points for gene silencing.

#### Correlation analysis between lncRNA and HSPs

qRT-PCR was used to assess the expression levels of the relevant HSP genes (*GB45913*, *GB47475*, *GB45910*, *GB50609*, *GB45495*, *GB45912*, *GB40976*) in honeybees after treatment with the optimal silencing fragment at various time points.

### Statistical analysis

PCR data were analyzed using a one-way analysis of variance to detect the homogeneity of variances, followed by Student’s *t*-test (PASS 18.0 software; https://www.ncss.com/). Data are shown as the mean ± standard error. *P* < 0.05 was considered statistically significant. The results were plotted using GraphPad Prism 8.0 (GraphPad Inc., La Jolla, CA, USA).

## Results

### Bioinformatics analysis of lncRNA

#### Identification and characterization of lncRNAs

**Fig. 1:**
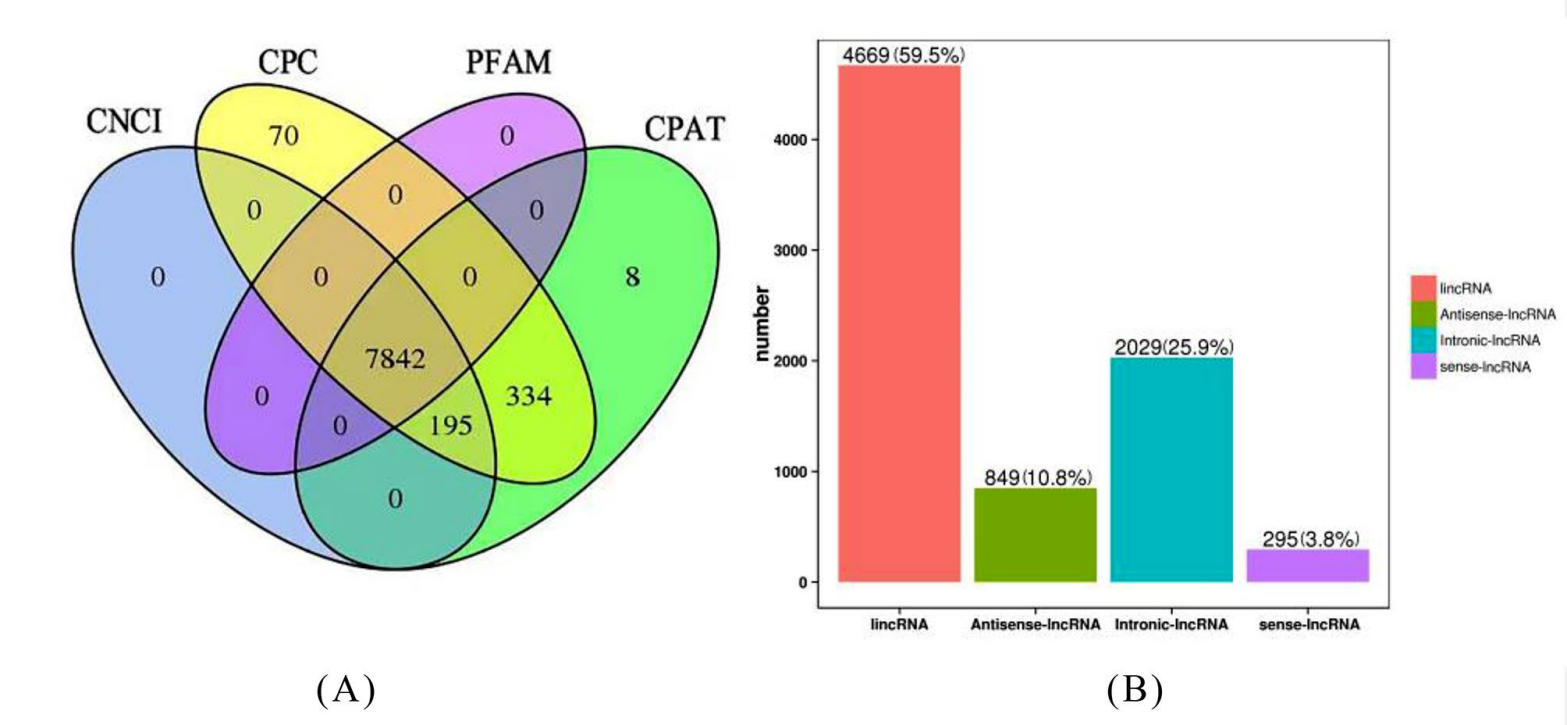
Identification and type distribution of long non-coding RNAs (lncRNAs). (**A**) Number of lncRNAs obtained by using CNCI, CPC, PFAM, and CPAT identification methods (**B**) Distribution of different types of lncRNAs. The y-axis represents the corresponding number of LncRNA. The X-axis represents the category of lncRNA

To identify and explore heat-responsive lncRNAs in worker bees, we identified lncRNAs from the sequence reads. Six individual cDNA libraries from the 25℃ (CK1, CK2, and CK3) and 45℃ (HT1, HT2, and HT3) groups were constructed. After filtering and screening, approximately 66-146 million clean reads were obtained per library and more than 63.33% of clean reads were mapped to the reference genome (Table 1). Q30 values ranged from 93.78 to 94.67%, indicating the credibility of the RNA-seq data. Overall, 7,842 lncRNAs were identified as lncRNAs after a series of strict screening pipelines using six libraries (Fig. 1A). The lncRNAs were subdivided into different categories according to their location. The majority of lncRNAs were lincRNAs (4699), antisense-lncRNAs (849), intronic-lncRNAs (2029), and sense lncRNAs (295) (Fig. 1B).

**Table 1:** LncRNA sequencing data compared to reference genomes

Samples	Total reads	Mapped reads	Multiple mapped reads	Q30 (%)
CK1	11,20,89,308	7,86,83,569 -70.20%	1,11,81,953 -9.98%	94.67
CK2	9,10,42,338	5,88,82,234 -64.68%	33,85,726 -3.72%	94.36
CK3	14,66,44,058	9,37,05,599 -63.90%	13,42,323 -0.92%	93.94
HT1	6,69,09,298	4,55,95,772 -68.15%	5,93,403 -0.89%	93.78
HT2	10,91,33,198	7,14,31,778 -65.45%	49,06,244 -4.50%	94.09
HT3	11,21,79,790	7,10,48,924 -63.33%	9,14,592 -0.82%	94.2

Note: Total Reads: The Number of Clean Reads; Mapped Reads: The Number of reads aligned to the reference genome and the percentage of Clean Reads; Uniq Mapped Reads: The Number of reads aligned uniquely to the reference genome and the percentage of Clean Reads; Q30 (%): The percentage of bases in Clean Data with a quality value greater than or equal to Q30.

**Fig. 2:**
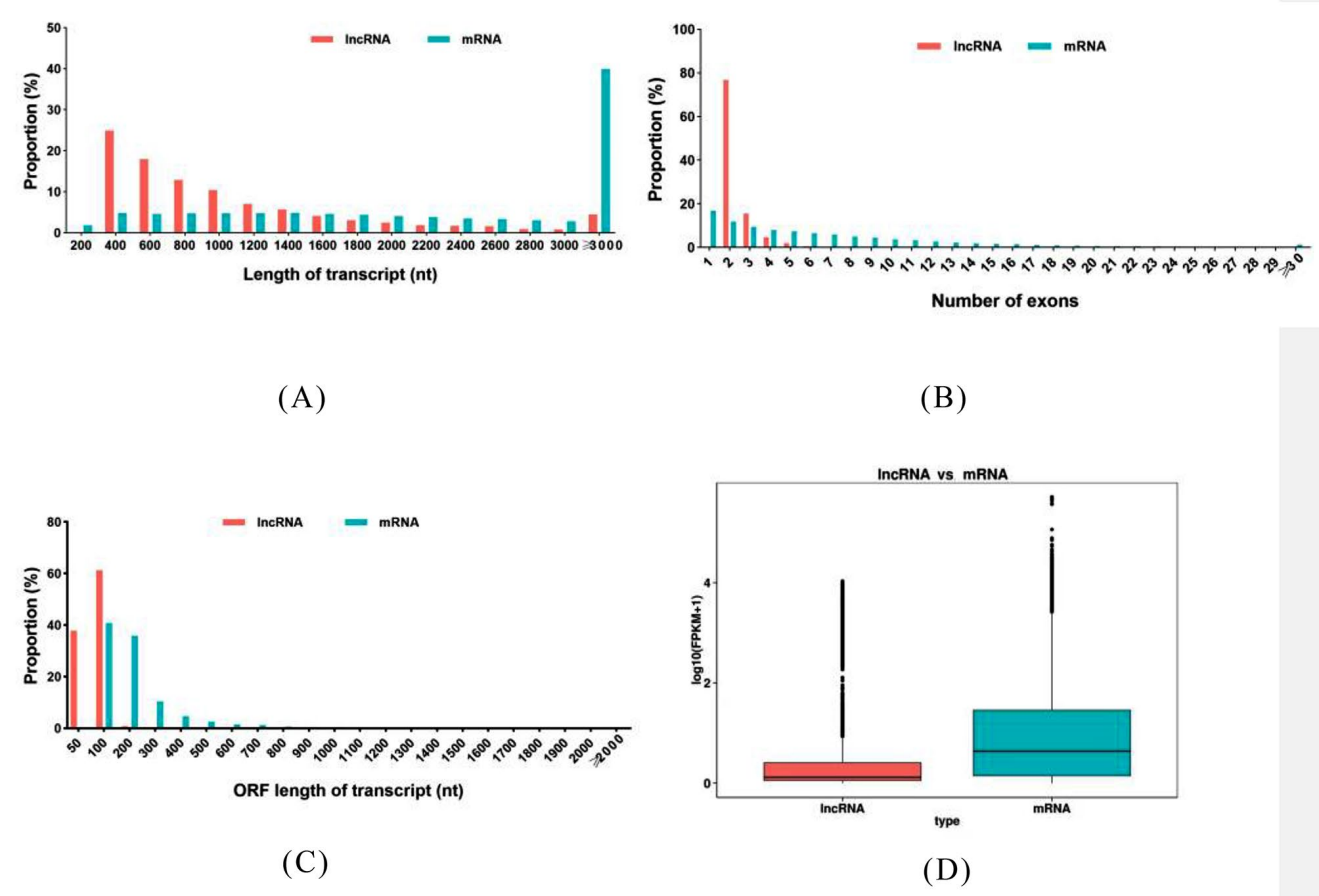
Comparative analysis of long non-coding RNAs (lncRNAs) and mRNA. (**A**) Length distribution. (**B**) Number of exons. (**C**) Open reading frame (ORF) length distribution between lncRNAs and mRNAs. (**D**) Expression levels

To further characterize the features of the identified lncRNAs in honeybees, we compared them with those of protein-coding mRNAs (Fig. 2). Structural analysis revealed that the overall expression levels of lncRNAs were lower than those of mRNA. The length of lncRNAs was in the range of 400-1,600 nt, markedly fewer lncRNAs were > 3,000 nt than were mRNAs. Most lncRNAs contained two exons, which were generally fewer than those of mRNA. Moreover, most lncRNAs had significantly shorter open reading frames (ORFs), ranging from 50 to 100 bp. Structural analysis showed that the overall expression abundance, exon number, and ORFs of lncRNAs were lower than those of the mRNA. Regardless of whether the samples were heat treated, the expression of lncRNAs was notably lower than that of mRNAs.

#### Differentially expressed genes

**Fig. 3:**
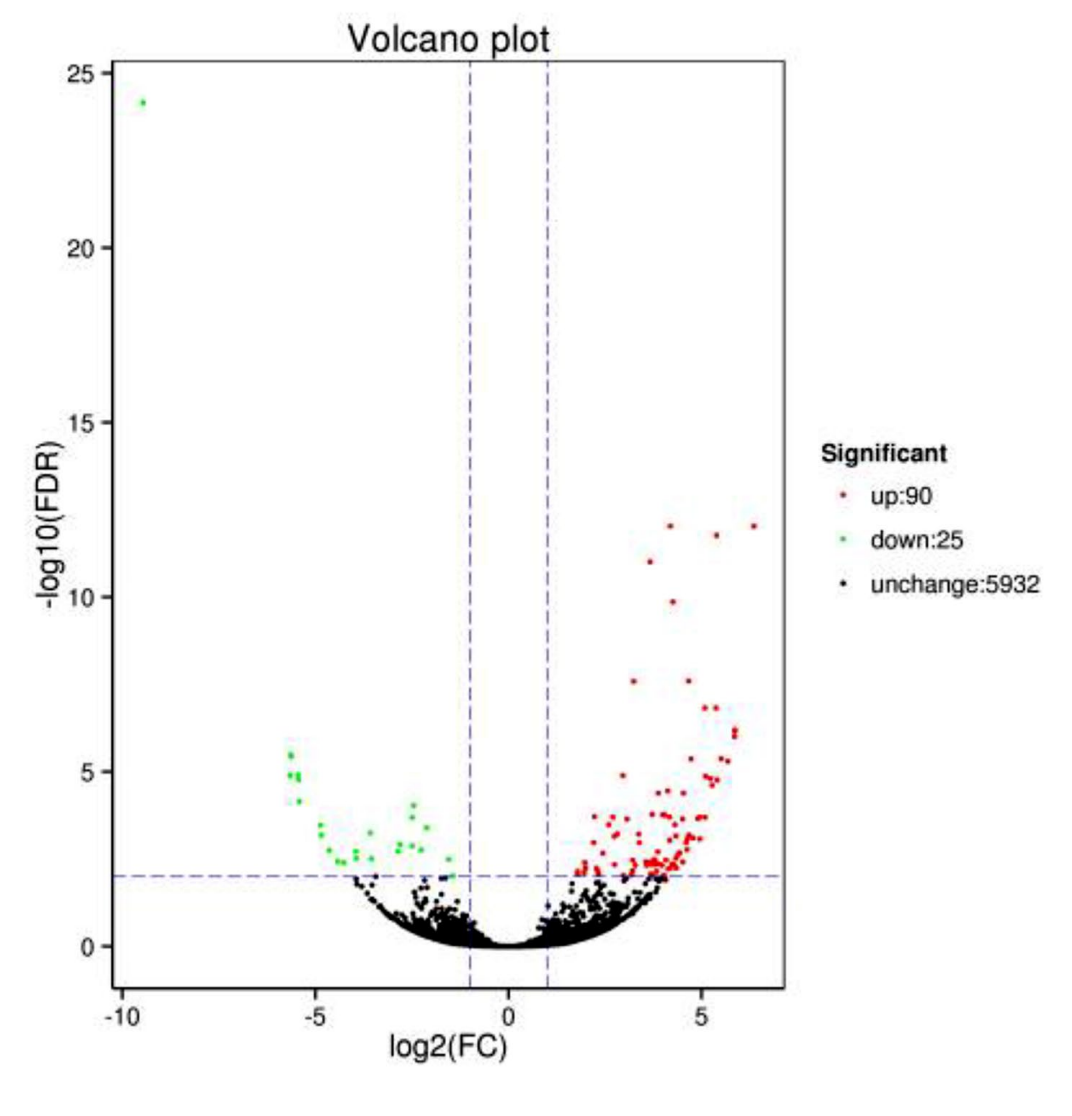
Differentially expressed long non-coding RNAs (lncRNAs) between normal conditions (CK) and high-temperature conditions (HT). Each point represents a single gene. Red represents the upregulation of lncRNAs, green represents the downregulation of lncRNAs, and black represents insignificant differences in lncRNAs

Using |fold change| ≥ 2 and FDR < 0.05 as screening criteria, 115 DELs (90 upregulated DELs and 25 downregulated DELs) were identified in the CK vs. HT (Fig. 3). Expression levels of most DELs were increased. The heat maps of these genes are shown in Supplementary Table 3.

#### GO and KEGG analysis of target genes

lncRNAs regulate proximal and distal protein-coding genes via cis- and trans-acting modes. We predicted the potential targets via cis- and trans-regulation. Therefore, we screened proximal protein-coding genes within 100 kb of lncRNAs as target genes for cis activity, resulting in the prediction of a total of 619 target genes. For the trans-regulation of lncRNAs, 1,030 target genes were predicted.

Overall, 315 significantly enriched GO terms (*p*-value < 0.05) were found in the CK and HT groups. In the GO analysis, genes were classified based on their functions, including biological processes (BP), molecular functions (MF), and cellular components (CC). The top three most significantly enriched GO categories are shown in Table 2. Based on the predefined significance level, the significantly enriched GO pathways were the following: in BP, the pathways with the most enriched DEGs included “cellular process”, “single-organism process” and “biological regulation”. In CC, the pathways were “cell”, “cell part”, and “organelle”. In MF, the pathways are “binding,” “catalytic activity” and “signal transducer activity.”

**Table 2:** GO classify of LncRNA target gene

GO classification 1	GO classification 2	Target genes of all lncRNAs	Target genes of DE lncRNA
Total gene		8944	1569
Biological process	cellular process	6148	691
	single-organism process	5242	626
	biological regulation	4356	485
Cellular component	cell	6008	612
	organelle	4907	483
	cell part	6007	612
Molecular function	binding	5833	654
	catalytic activity	3476	392
	transporter activity	700	82

Note: GO classification 1: First-level classification; GO classification 2: Second-level classification.

**Fig. 4:**
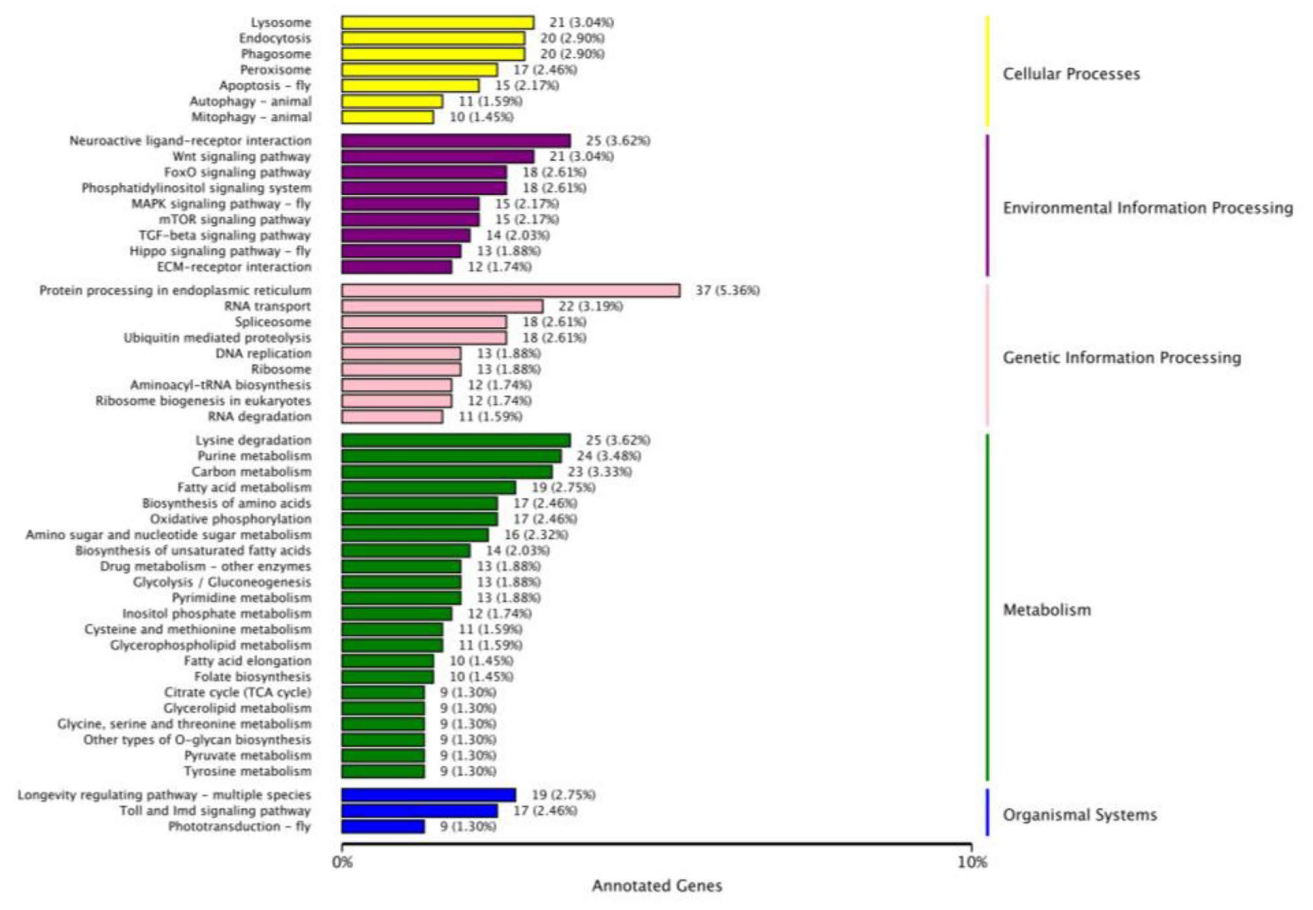
KEGG enrichment analysis of differentially expressed long non-coding RNA (lncRNA) target gene. The numbers following the bar charts indicate the number of target genes of lncRNAs in this pathway

In the KEGG analysis of CK vs. HT (Fig. 4), the most significantly enriched KEGG pathways were associated with metabolic processing (e.g., amino acid, carbon, oxidative phosphorylation, and glycolysis/gluconeogenesis). Furthermore, many enriched pathways were involved in genetic information processing (e.g., RNA transport, DNA replication, and protein processing in the endoplasmic reticulum and ribosomes). In addition, many enriched pathways were involved in environmental information processing (e.g., neuroactive ligand–receptor interaction, WNT pathway, phosphatidylinositol signaling system, and MAPK pathway).

#### Validation of DELs

**Fig. 5:**
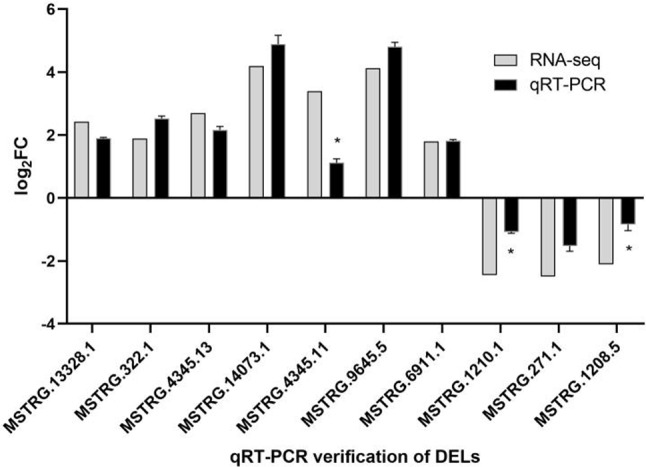
Validation of long non-coding RNAs (lncRNAs) selected using quantitative real-time polymerase chain reaction. Data are presented as the mean ± SE. “*” indicates *p* < 0.05

To validate the accuracy of the transcriptome results, qRT-PCR was performed to examine the expression levels of differentially expressed genes in the temperature-treated groups. qRT-PCR analysis of these genes aligned with the transcriptome data, indicating the authenticity and reliability of the transcriptome results (Fig. 5). *MSTRG.4345.11*, *MSTRG.1210.1* and *MSTRG.1208.5* showed significant differences in expression levels between methods. The difference in fold change calculated by RNA-seq and PCR may be due to the differences in their technical principles and data processing methods. In the transcriptome experiment, *MSTRG.9645.5* (FPKM > 300) showed the highest expression level, which was confirmed by qRT-PCR analysis. Therefore, this gene was selected as the target for interference.

### Interference result

#### Subcellular localization prediction and target analysis of MSTRG.9645.5

**Fig. 6:**
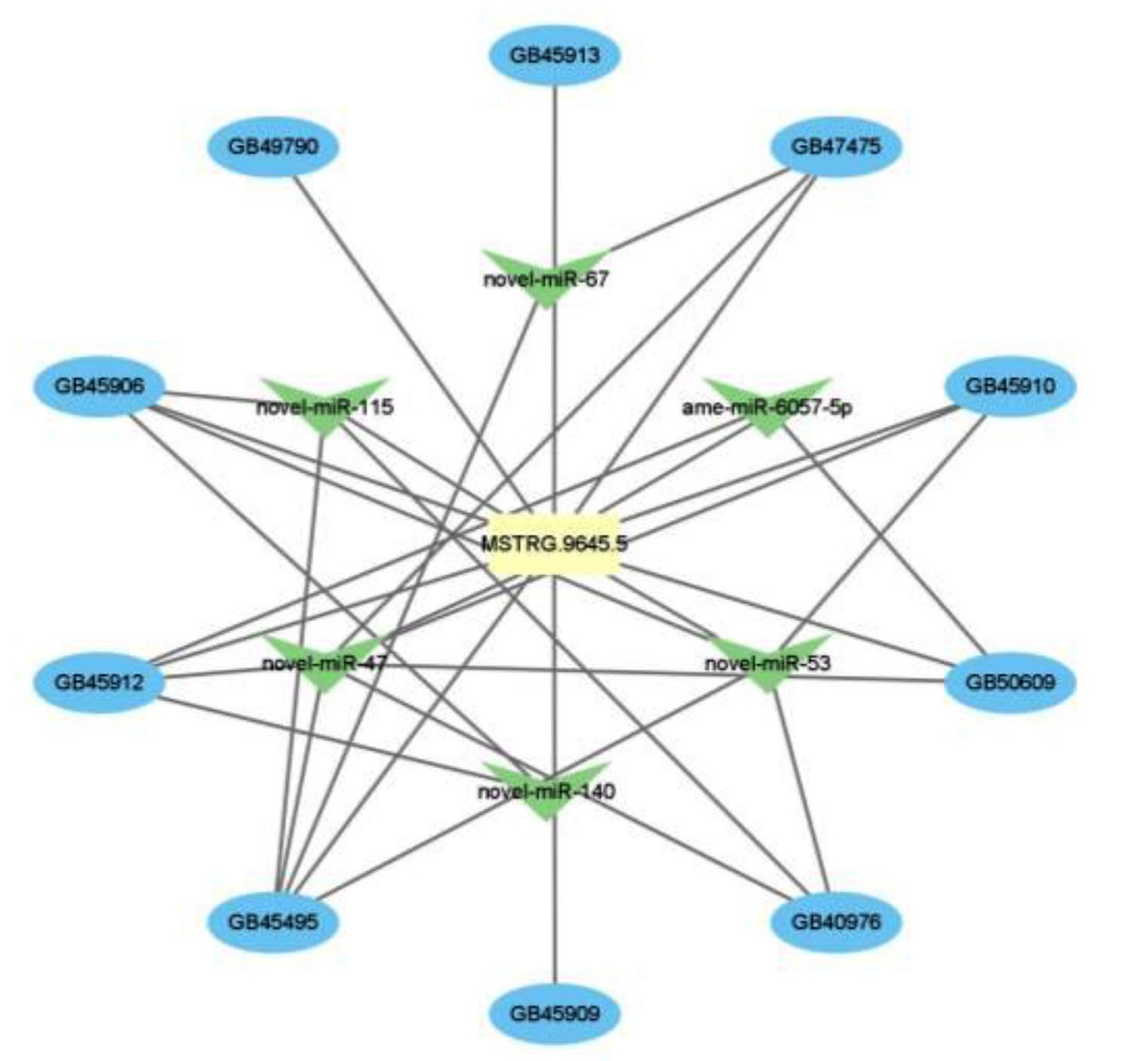
Regulatory network between *MSTRG.9645.5*, target differentially expressed (DE) miRNAs and target DE mRNAs.

*MSTRG.9645.5* was present in both the nucleus and cytoplasm, with its predominant presence in the nucleus. This suggests that *MSTRG.9645.5* may function as a competing endogenous RNA (ceRNA) [10, 47]. Additionally, *MSTRG.9645.5* predicted 88 target miRNAs, including six differentially expressed (DE) miRNAs. Moreover, the expression level of *ame-miR-6057-5p* (log_2_ FC = 1.23, *p* < 0.05) was found to be significantly up-regulated in the high-temperature group and the expression level of *novel-miR-53* (log_2_ FC=-1.36, *p* < 0.05) was found to be significantly up-regulated. These results support the notion that *MSTRG.9645.5* likely exerts a regulatory role as a ceRNA, potentially through its interaction with miRNAs, forming a regulatory network, as depicted in Fig. 6.

#### Determining the optimal interference conditions for lncRNA

**Fig. 7:**
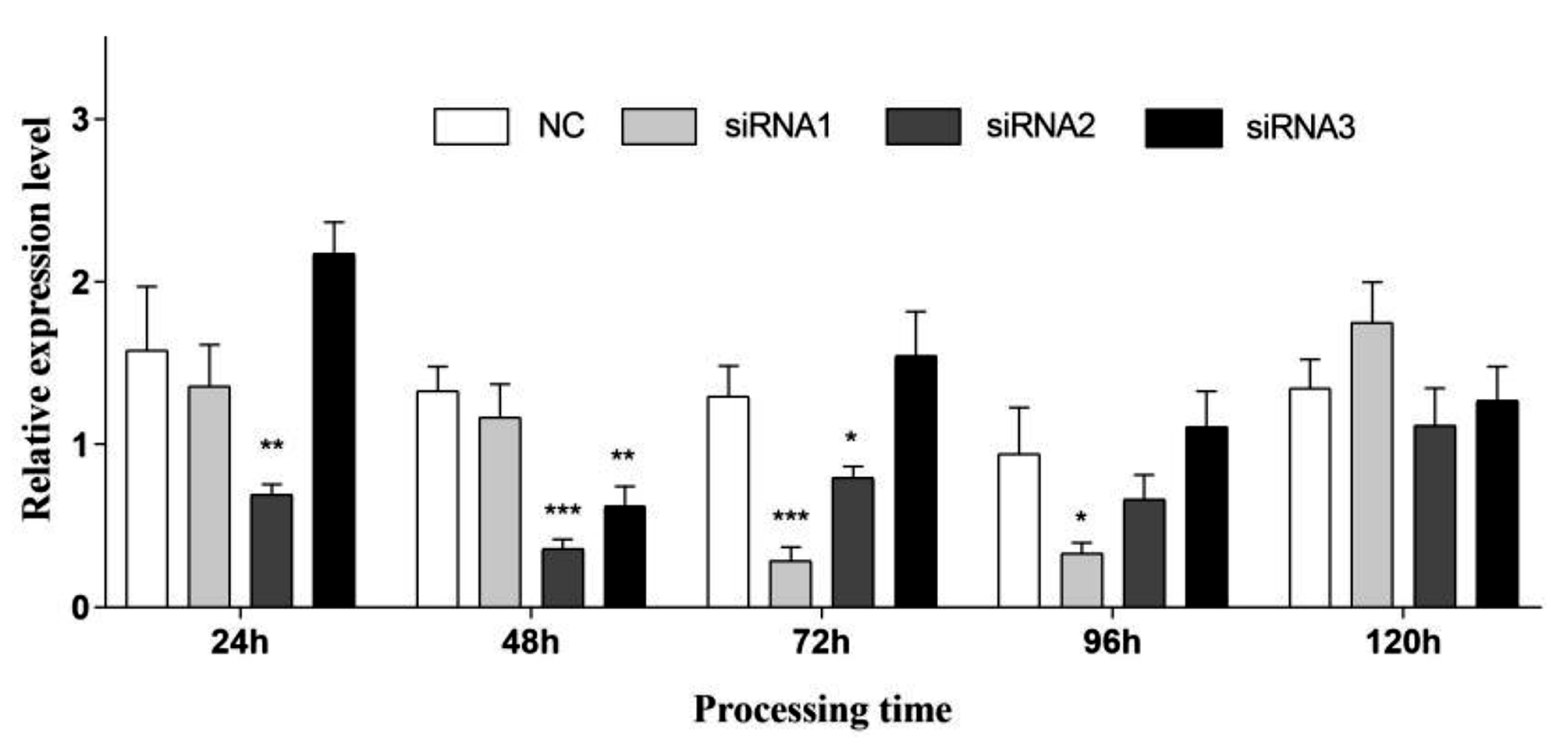
Screening for the best interfering fragment. “***” indicates *p* < 0.001, “**” indicates *p* < 0.01, “*” indicates *p* < 0.05

In the interference experiment, we designed three siRNAs and used feed administration to interfere with *MSTRG.9645.5* in honeybees. Subsequently, qRT-PCR was used to detect the expression levels of lncRNAs in the honeybee body within 1-5 days after siRNA feeding. The results showed that, in the siRNA1 group, a significant silencing effect was achieved at 72 and 96 h, reaching extremely significant (*p* < 0.001) at 72 h. In the siRNA2 group, there was a varying degree of decrease in lncRNA expression at 24, 48 and 72 h, reaching extremely significant (*p* < 0.001) at 48 h. In contrast, siRNA3 did not show a significant decreasing trend after silencing but exhibited a significant decrease (*p* < 0.01) at 48 h. After 120 h, the interference effects of all three interference fragments disappeared (Fig. 7).

#### The changes in the expression of the associated genes after interference

**Fig. 8:**
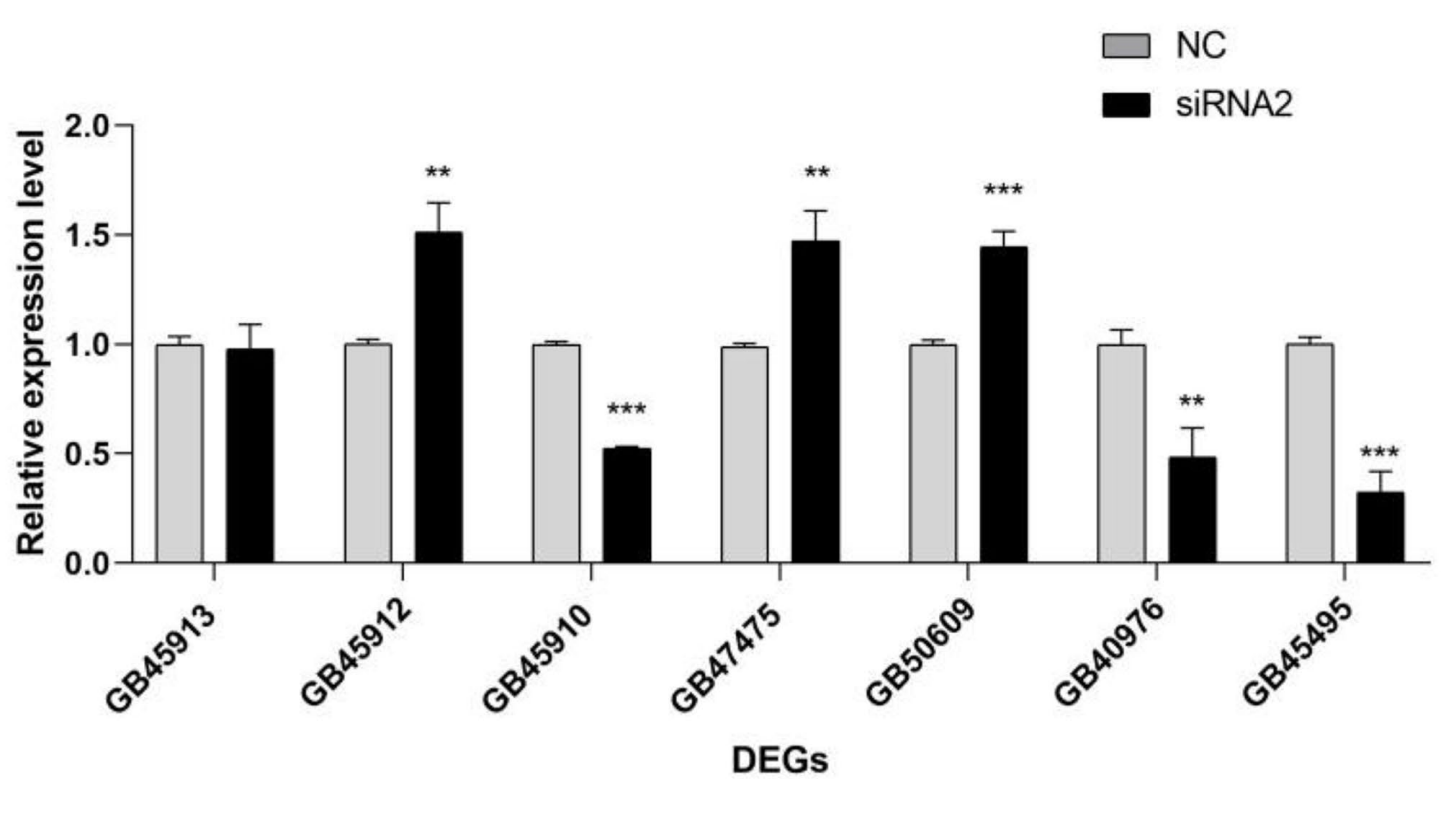
Relative expression levels of heat shock protein genes after long non-coding RNA (lncRNA) silencing. “***” *p* indicates < 0.001, “**” indicates *p* < 0.01

After interfering with the lncRNA (*MSTRG.9645.5*), qRT-PCR was used to examine the expression levels of seven heat-related DEGs (PCC > 0.99). The results showed that, among these seven genes (Fig. 8), the expression of three genes showed extremely significant differences (*p* < 0.001), while the expression of another three genes also showed highly significant differences (*p* < 0.01), and that of the remaining one gene showing no significant change. Among them, three genes were upregulated (*L(2)efl* family genes *GB45912* and *GB47475*,* Hsp70Ab-like* *GB50609*), and three genes were downregulated (*L(2)efl GB45910*, *Hsp90 GB40976*, *Hsp83 GB45495*).

## Discussion

LncRNAs are widely present in different species and play fundamental roles in abiotic stress responses [48, 49]. A considerable number of lncRNAs have been discovered to respond to various abiotic stresses in insects [15, 50]. The lncRNA-heat shock RNA omega (*hsrω*) is a stress-induced and developmentally expressed lncRNA, which plays a critical regulatory role in cellular aging and thermal stress response in *Drosophila* [51, 52]. However, the potential role of heat-shock related (or heat-shock associated) lncRNAs in mediating thermal stress response in honey bees remains unknown. Extreme heat events are expected to impact pollinators negatively [53]. Therefore, understanding and mitigating the effects of climate change on pollinators is vital for maintaining ecosystem health and for promoting sustainable agricultural development.

Bees exhibit the ability to adapt to high-temperature environments through a regulatory network composed of HSPs, transcription factors, and non-coding RNAs [4, 15]. These molecules help cells to counteract protein inactivation and oxidative stress caused by high temperatures, thereby maintaining cellular stability and enhancing heat tolerance [4, 15, 54]. This study systematically identified 7,842 potential lncRNAs in worker bees, providing abundance resources for future studies. The general characteristics of lncRNAs in worker bees subjected to heat treatment have been described in detail in this study. The majority of lncRNAs had a length of < 1,400 nt and contained two exons (Fig. 2), which was in accordance with the findings of previous studies on other species [55]. The number of identified lncRNAs in our study was greater than that identified in *Apis mellifera* [19], which may be partially due to variation in insect subspecies, genotypic milieu, patrilines, the influence of sequencing depth, or experimental conditions.

LncRNAs are involved in essential biological processes, such as imprinting, gene regulation, and dosage compensation [56, 57]. In this study, we found several highly-expressed lncRNAs responding to high-temperature stress in honeybees, and potentially participating in this response by regulating the levels of heat shock-related genes. Whether and how these heat-shock related genes contribute to heat stress response in bees is not within the scope of this study. First, lncRNAs (*MSTRG.9645.5* and *MSTRG.14073.1*) were highly expressed in the HT group and both targeted the genes (HSPs and HSP-related genes) in trans-regulation. The expression levels of HSP-encoding genes are significantly increased in honeybees exposed to high-temperature environments [4, 5]. Additionally, the expression levels of lncRNAs were consistent with their mRNA expression levels. This phenomenon may indicate that lncRNAs play an important role in responding to high-temperature stress. The upregulation of both lncRNAs and their target gene mRNAs suggests that lncRNAs may be involved in regulating the adaptive response of organisms to high temperatures by controlling the expression of their target genes. This regulation may occur through promoting or inhibiting the transcription or translation of target genes, thereby influencing the physiological and biochemical adaptations of honeybees to high-temperature stress.

Furthermore, we found that the lncRNA *MSTRG.11762.4* was downregulated, whereas its target gene *L(2)efl* (lethal(2) essential for life, *GB45910*) in the trans group was upregulated in the HT group. *L(2)efl* is responsive to a broad array of stressors and is a potentially promising biomarker of honeybee stress [54]. Interestingly, the encoded HSPs were enriched in the “protein processing in endoplasmic reticulum” and “longevity regulating pathway-multiple species” pathways (Fig. S1). Further experimental studies are required to confirm these mechanisms.

Predicting the target genes of lncRNAs, performing functional annotation and enrichment analyses are important approaches for studying lncRNA functions [58]. In this study, combined GO and KEGG enrichment analyses revealed that the DEL target genes were related to various aspects of bee growth and development, including carbon metabolism, amino acid metabolism, lipid metabolism, ribosomes, starch and sugar metabolism, and hormone signaling pathways. These findings indicate the crucial role of lncRNAs in the regulating of fundamental metabolic and regulatory mechanisms involved in bee growth and development.

Specifically, GO and KEGG enrichment analysis revealed a significant enrichment of DEGs involved in signaling transduction-related pathways, such as WNT, TGF-*β*, and MAPK pathways. Further analysis demonstrated the involvement of various signaling molecules, membrane receptors, transcription factors, and nucleic acid-binding proteins in these pathways, all closely associated with cell growth, differentiation, apoptosis, metabolism, immune response, and other biological processes [59–61]. Previous studies have found that some lncRNAs can modulate cell signal transduction and biological processes by influencing downstream components of the MAPK pathway [47]. LncRNAs were involved in the hippo and TGF-*β* signaling pathways [21]. In summary, these analysis indicate that lncRNAs may affect the relevant reactions of honeybees to heat stress by regulating components of signal transduction pathways and signaling molecules, such as kinases and transcription factors, or by mediating the regulation of signaling molecules, such as mRNA-binding proteins.

Previous studies have shown that dsRNA can silence lncRNAs in insects, such as *Drosophila* [62], silkworms [63], and *Apis cerana cerana* [64], through injection or feeding. In our study, in the siRNA1 group, the expression of *MSTRG.9645.5* was significantly downregulated (by 77.51%) after 72 h, whereas in the siRNA2 group, it was downregulated by 72.93% after 48 h. Thus, we revealed that specific siRNAs, delivered through feeding, effectively silenced the expression of *MSTRG.9645.5* in honeybees. The study on siRNA-mediated silencing of bee lncRNAs provided an experimental basis for studying lncRNA function in *A. mellifera* worker bees and possibly other bee species.

*L(2)efl* is upregulated in response to proteotoxic stress, and its encoded sHSP protects cells by maintaining improperly folded proteins in a soluble state, preventing the formation of harmful aggregates and mitigating protein damage-induced cell death and disease [54]. In this study, downregulation of the target gene *L(2)efl* (*GB45910*) and upregulation of the target genes *L(2)efl* (*GB45912*, *GB47475*) were observed after silencing *MSTRG.9645.5*, indicating that *MSTRG.9645.5* may regulate the expression of *L(2)efl*, thereby influencing the protein folding process mediated by sHSPs.

HSPs play an important role in response to changes in both the intracellular and extracellular environments, maintaining protein homeostasis, and preserving cellular functions [4, 5]. Their expression is often upregulated when cells are subjected to heat shock or other stressful stimuli, thereby playing a protective role in preventing cell damage. In this study, downregulation of the target genes *HSP90* (*GB40976*) and *HSP83* (*GB45495*), and upregulation of the target gene *HSP70* (*GB50609*) were observed after silencing *MSTRG.9645.5*, indicating that *MSTRG.9645.5* may regulate the expression of HSPs.

Interference with lncRNAs can result in either upregulation or downregulation of target gene expression, depending on their specific function and interaction, highlighting the need for further research to understand their regulatory mechanisms. Future studies will focus on predicting the targeting of lncRNA-miRNAs and studying HSP genes through RNA interference to gain further understanding of the ceRNA mechanism of lncRNA regulations in response to heat stress.

## Conclusions

Elevated temperatures negatively affect honeybees. LncRNAs play critical roles in abiotic stress responses. We here generated the expression profiles of lncRNAs in worker bees under heat stress using deep RNA-seq, and identified 115 DELs (90 upregulated and 25 downregulated). Bioinformatics analysis showed that the target genes of DELs were involved in important biological processes, such as metabolism, protein folding, response to stress, and signal transduction pathways. One significant finding of our study was the effective silencing of LncRNA *MSTRG.9645.5* in honeybees through siRNA feeding. LncRNA *MSTRG.9645.5* may regulate the response mechanisms to heat stress in honeybees by targeting HSP mRNAs. The critical lncRNAs identified in our study provide valuable information for understanding heat-responsive lncRNAs in worker bees and represent a rich resource for further investigation of the biological functions of lncRNAs in insects.

### Electronic supplementary material

Below is the link to the electronic supplementary material.

Supplementary Material 1

Supplementary Material 2

## Data Availability

The sequencing data are available in the BioProject database (PRJNA880001) of the NCBI system.(https://www.ncbi.nlm.nih.gov/bioproject/?term=PRJNA880001).
