# Supplementary material for: Regulatory roles of long non-coding RNAs in short-term heat stress in adult worker bees
Source: BMC Genomics. 2024 May 22;25:506. doi: 10.1186/s12864-024-10399-8 (PMC11110378; doi:10.1186/s12864-024-10399-8)
Supplement: Supplementary file 1 — Supplementary Material 1 [file 12864_2024_10399_MOESM1_ESM.doc]

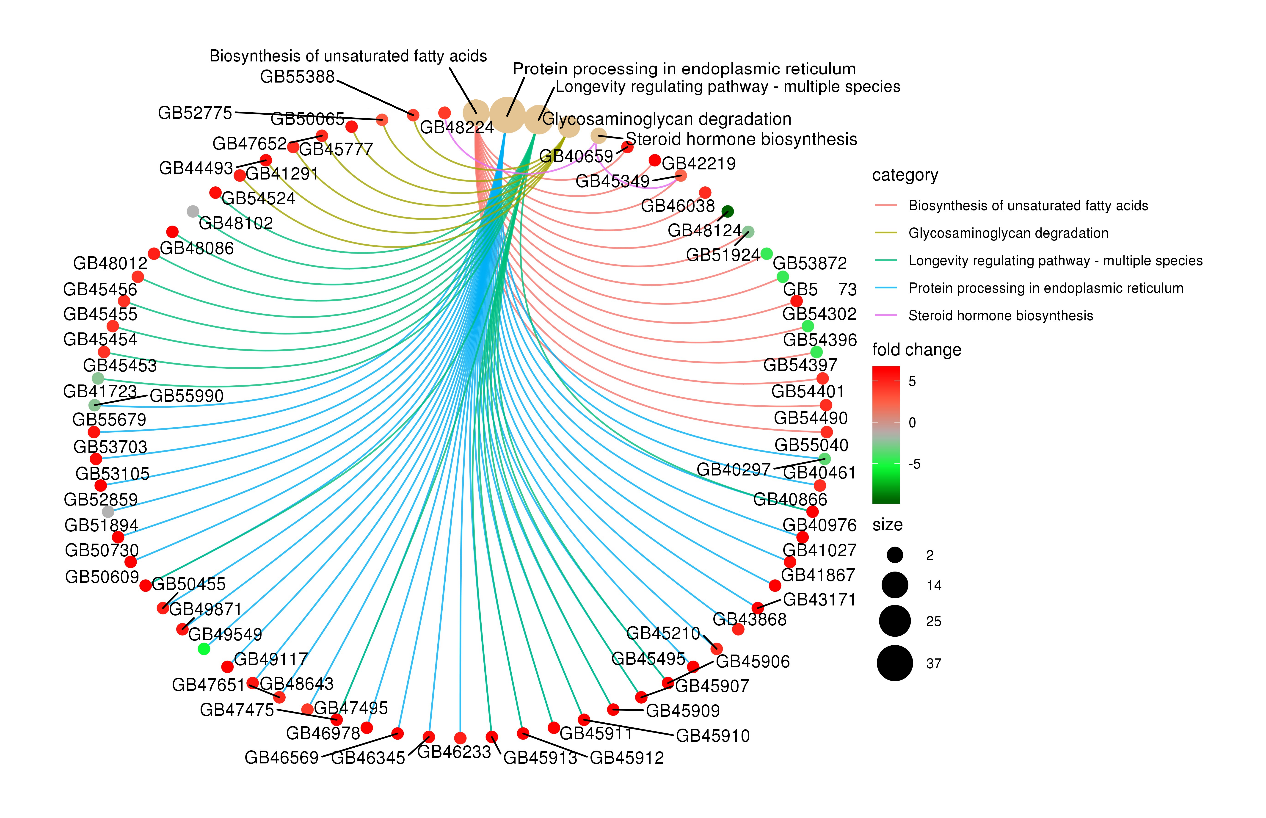


**Supplementary figure 1 Analysis of target genes of lncRNAs and their enriched in the top-5 pathway.** red color represents up-regulated differentially expressed genes, green color represents down-regulated differentially expressed genes, and the intensity of the color indicates the magnitude of differential expression.
